# Dissociative Symptoms and Disorders in Patients With Bipolar Disorders: A Scoping Review

**DOI:** 10.3389/fpsyt.2022.925983

**Published:** 2022-05-26

**Authors:** Ravi Philip Rajkumar

**Affiliations:** Department of Psychiatry, Jawaharlal Institute of Postgraduate Medical Education and Research, Pondicherry, India

**Keywords:** bipolar disorders, dissociative disorders, depression, depersonalization, derealization, comorbidity

## Abstract

Dissociative disorders are an important group of trauma-related disorders associated with significant disability. The co-occurrence of dissociative disorders (DD) and symptoms (DS) in bipolar disorder has been relatively understudied, but there is some evidence that this comorbidity may have significant mechanistic and clinical implications. This paper presents the results of a scoping review of the frequency and correlates of DS and DD in bipolar disorder. Based on the available evidence, DS/DD are more common in bipolar disorder than in healthy controls or in unipolar depression, are related to childhood trauma, and are associated with psychotic symptoms, suicide attempts, and a poorer response to treatment in patients with bipolar disorder. The implications of these findings, and possible mechanistic pathways underlying them, are discussed based on the current literature. Clinicians should be aware of the frequent occurrence of significant DS or DD when treating patients with bipolar disorder. A tentative future research agenda for this field, based on clinical, risk factor-related and neurobiological considerations, is outlined.

## Introduction

Bipolar disorders are a group of mental illnesses characterized by recurrent episodes of elevated and depressed mood, associated with significant levels of morbidity and an elevated mortality risk (1). Comorbidity with other psychiatric disorders is seen in over 50% of patients with BD, particularly with anxiety, attention-deficit/hyperactivity and substance use disorders (2). The presence of comorbid diagnoses in BD is associated with poorer treatment response and a more severe illness course; patients with these diagnoses often require more intensive or complex treatment regimens (3–6).

Dissociative disorders (DD), which are characterized by disruption or discontinuity in the integration of one's consciousness, memory, identity and behavior, are associated with risks of hospitalization, self-injury and suicide comparable to BD (7). Though the comorbidity of DD and BD has been relatively under-studied (8), there is evidence from the literature of several potential clinical and mechanistic links between them. BD and DD appear to share a genetic substrate to some extent (9, 10) and the onset of symptoms of DD may be a herald of subsequent BD in adolescents (11, 12). DD may be underdiagnosed in patients with BD because of diagnostic criteria that do not allow DD to be diagnosed in the presence of depression (13), confusion arising from similar symptoms (14), or a reluctance to diagnose DD among mental health professionals (15). A further problem is posed by patients with BD who have features of dissociation that are clinically significant, but do not fulfill criteria for DD; these are referred to as “pathological dissociation” or “dissociative symptoms” (DS).

The present of DD or DS in BD raises several important questions. How frequent and severe is this comorbidity? What is the impact of DD/DS on the clinical features and prognosis of BD? Are there any specific environmental risk factors, such as childhood adversity, that are associated with the presence of DD/DS? Are DD/DS in BD associated with specific genetic factors or other biomarkers? The current scoping review aims to address these questions in a preliminary manner.

## Methodology

Given the lack of a single specific question and the paucity of literature in this area, a scoping review was carried out instead of a systematic review (16). This review was carried out in accordance with the PRISMA guideline for scoping reviews (PRISMA-ScR) (17). The PubMed, Scopus and ScienceDirect databases were searched using combinations of the key words “bipolar disorder”, “bipolar disorders”, “bipolar spectrum”, “bipolar I disorder”, “bipolar II disorder” in association with “dissociative disorders”, “dissociative amnesia”, “dissociative identity disorder”, “depersonalization”, “derealization”, “dissociative symptoms”, “pathological dissociation”. All studies published up to April 15, 2022 were included in this review. Studies were included if they measured the frequency and/or correlates of the presence of DD/DS in BD, with or without the inclusion of comparator groups. Any study that provided information on the frequency, severity, clinical impact, association with environmental risk factors, or neurobiological correlates of DS/DD in patients with bipolar disorder was included in this review. Case reports/series, editorials, commentaries and general review articles were excluded.

**Figure 1:**
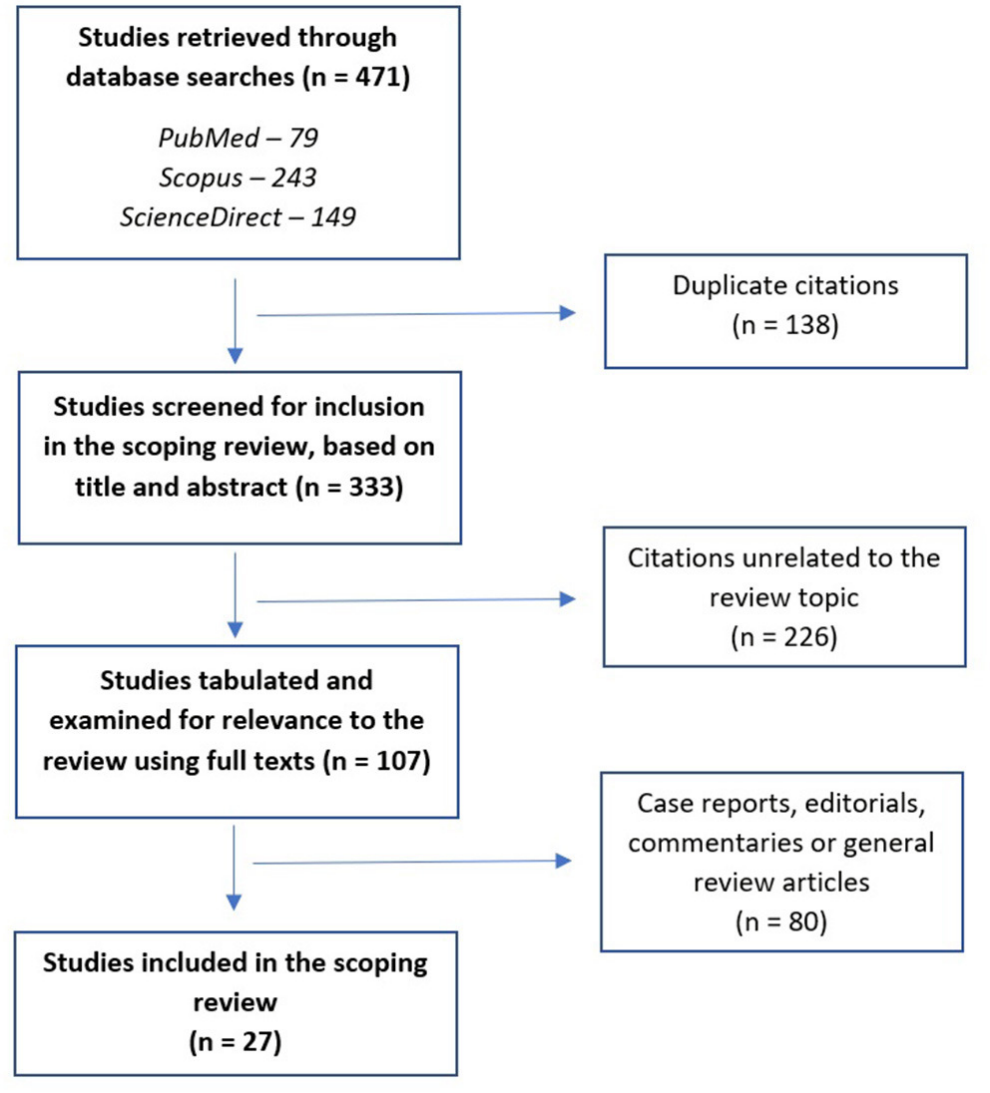
PRISMA-ScR flow diagram for the current scoping review.

A total of 471 citations were retrieved; after removal of duplicates, 333 citations were screened; after exclusion of 226 unrelated abstracts, 107 citations were tabulated and their full text was examined for relevance. Of these, 27 were included in the final review (18–44). This process is illustrated through a PRISMA-ScR flow diagram in Figure 1.

Following tabulation, study results were sorted thematically according to the objectives of this review, as follows:

- Frequency of comorbid DD in BD or vice versa
- Frequency of significant DS in BD
- Comparison of DD/DS in BD compared to other groups (major depression, other psychiatric diagnoses, healthy controls)
- Associations with BD subtype (e.g. type I vs type II)
- Associations with BD course (e.g., age at onset, number of episodes)
- Associations with BD symptomatology (e.g., mixed features, psychotic symptoms, suicide attempts)
- Associations with BD outcome (e.g., treatment response, disability, quality of life)
- Associations with other comorbidities in BD (e.g., anxiety disorders, substance use disorders)
- Associations with environmental factors in BD (e.g., childhood trauma, current stressors)
- Associations with other psychological variables (e.g., temperament, personality traits)
- Associations with neuropsychological deficits in BD (e.g., attention or memory deficits)
- Associations with genetic or other biological markers (e.g., specific genetic polymorphisms, levels of hormones or inflammatory markers)

The same schema was followed when reporting the results.

## Results

A complete description of the included studies is provided, in chronological order, in Table 1.

**Table 1:** Studies examining the association between dissociative symptoms or disorders and bipolar disorder, with a summary of their key findings.

**References**	**Study population and sample size**	**Variables or outcomes studied**	**Results**
Nijenhuis et al. (18)	Patients with BD (*n =* 41) and DD (*n =* 51)	DS severity across groups, rated using DES	DES scores higher in DD than in BD; significant DS in 10% of BD patients.
Nijenhuis et al. (19)	Patients with BD (*n =* 23), DD (*n =* 44), somatoform disorders (*n =* 47), eating disorders (*n =* 50), and other psychiatric diagnoses (*n =* 45)	DS severity across groups, rated using DES; somatoform DS severity across groups, rated using SDQ-20;	DES and SDQ-20 positively correlated; DES scores significantly lower in BD than in DD; DES comparable in BD and somatoform disorders; SDQ-20 significantly lower in BD than in somatoform disorders and DD.
Hlastala & McClellan (20)	Youth with psychotic BD (*n =* 22), schizophrenia (*n =* 27) and atypical psychosis (*n =* 20)	DS severity across groups, rated using DES	DES scores higher in atypical psychosis than in BD or schizophrenia.
Johnson et al. (21)	Community-dwelling adults (*n =* 658)	Prevalence and comorbidities of dissociative disorders as per DSM-IV	Diagnosis of dissociative disorder 2.4 times more likely in those with a mood disorder (unipolar depression or BD).
Oedegaard et al. (22)	In- and out-patients with BD-II (*n =* 24) and MDD (*n =* 41)	DS severity across groups, rated using DES; association with temperament and comorbid diagnoses.	DES scores higher in BD-II than in unipolar depression; cyclothymic temperament associated with higher DES scores; pathological dissociation associated with comorbid OCD
Savitz et al. (23)	Patients with BD-I (*n =* 31), BD-II (*n =* 16), and their first-degree relatives with MDD (*n =* 64), other psychiatric diagnoses (*n =* 17) or no psychiatric diagnosis (*n =* 50)	DS severity across groups, rating using DES; association with childhood trauma and polymorphisms of *BDNF, COMT, DAT, DRD4*, and *SERT* genes.	DES scores higher in BD-I and BD-II than in relatives with no or “other” diagnoses; significant interaction between *COMT* genotype and childhood trauma associated with DES score; additive effect of *BDNF* genotype on DES score.
Mula et al. (24)	Patients with BD-I (*n =* 43) and BD-II (*n =* 48), euthymic	DS severity rated using DES; depersonalization symptom severity using SCI-DER; association with temperament, illness course and comorbidities	DES and SCI-DER scores comparable in BD-I and BD-II; no association of DES or SCI-DER scores with temperament; DES and SCI-DER scores associated with earlier AAO; higher SCI-DER scores in BD with comorbid panic disorder.
Latalova et al. (25)	Patients with BD, euthymic (*n =* 23)	DS severity rated using DES; association with illness course, tests of attention, verbal fluency and executive function, and quality of life.	DES scores associated with higher number of manic episodes, higher mean mood stabilizer dosage, and lower quality of life in the “social activities” domain. No correlation between DES and cognition.
Mula et al. (26)	Patients with mood and anxiety disorders (*n =* 258) including BD-I (*n =* 43) and BD-II (*n =* 48)	Depersonalization symptom severity using SCI-DER; distinction between depersonalization and anhedonia	SCI-DER scores higher in BD than in unipolar depression; SCI-DER score associated with earlier AAO in BD.
Chien et al. (27)	College students (*n =* 2,731)	Psychiatric symptoms as per DSM-IV using the Adult Self Report Inventory-4	Significant positive correlation between symptoms of bipolar and dissociative disorders independent of gender.
Latalova et al. (28)	Patients with BD, euthymic (*n =* 41), healthy controls (*n =* 198)	DS severity rated using DES; association with demographic variables and illness course.	Significant dissociation (DES > 30) in 51.2% of BD patients; DES total and sub-scores significantly higher in BD than in controls; pathological dissociation associated with earlier AAO.
Weber et al. (29)	Discharge records of patients with BD (*n =* 27,054) compared to those with other diagnoses (*n =* 2,325,247)	Presence of comorbid “anxiety, dissociative and somatoform disorders” in BD; comparison of morbidity between BD and those with other diagnoses	“Anxiety, dissociative and somatoform disorders” identified in 11.4% of BD discharge records; “anxiety, dissociative and somatoform disorders” associated with 2.8 times greater morbidity in BD than in those with other diagnoses.
Souery et al. (30)	Patients with BD-I (*n =* 104), BD-II (*n =* 64), and MDD (*n =* 123; 53 with family history of BD)	Depersonalization symptom severity across groups, rated using item 19 of HAM-D	Depersonalization symptom severity greater in BD-I than in BD-II or MDD.
Macri et al. (31)	Outpatients with BD (*n =* 17), MDD (*n =* 18), anxiety disorders (*n =* 32), adjustment disorders (*n =* 11) and somatoform disorders (*n =* 5)	DS severity rated using DES; association with severity of depression and psychopathological domains on SCL-90-R	No association between DS severity and depression severity; total DES score positively correlated with all nine domains of SCL-90-R and with overall global severity of illness across diagnoses.
Dorahy et al. (32)	Patients with dissociative disorder (*n =* 39), complex PTSD (*n =* 13) and mood disorders (*n =* 21)	DS severity across groups, rated using DES	Dissociative symptoms higher in the dissociative disorder group than in the complex PTSD or mood disorder groups.
Eryilmaz et al. (33)	Patients with BD-II, euthymic (*n =* 33), healthy controls (*n =* 50)	DS severity across groups, rated using DES; association with childhood trauma and obsessive-compulsive symptoms	Significant dissociation (DES > 30) in 15.2% of BD-II patients; dissociative symptoms higher in BD-II than controls; DES correlated with scores for childhood trauma and OCD symptoms in BD-II
Hariri et al. (34)	Patients with BD, euthymic (*n =* 200), healthy controls (*n =* 50)	DS severity across groups, rated using DES	Significant dissociation (DES > 30) in 19.5% of BD patients; depersonalization/amnesia symptoms associated with earlier AAO and longer duration of BD; absorption/identity symptoms associated with earlier AAO.
Yayla et al. (35)	Patients with conversion disorder (*n =* 54)	Prevalence of DSM-IV DD and comorbidities	27.8% of patients with DD had comorbid BD; BD more common in patients with DD.
Bayes et al. (36)	Patients with BD or borderline personality disorder (*n =* 226)	Depersonalization symptoms (self-reported)	Depersonalization symptoms more common in borderline personality disorder than in BD.
Yilmaz et al. (37)	Patients with BD-euthymic (*n =* 70), healthy controls (*n =* 70)	DS severity across groups as rated using DES; association with childhood trauma, illness course and alexithymia	DES scores higher in BD than in healthy controls; DES score significantly associated with episode frequency and alexithymia but not with childhood trauma.
Chatterjee et al. (38)	Patients with BD-depression (*n =* 35) and recurrent MDD (*n =* 36)	DS severity as rated using DES-II	DS more severe in BD than in unipolar depression; no correlation of DES-II with AAO, illness duration or number of episodes in BD.
Kefeli et al. (39)	Patients with BD-I-euthymic (*n =* 40), healthy controls (*n =* 40)	DS severity as rated using DES-II; association with number of manic and depressive episodes	Significant dissociation (DES > 30) in 20% of BD-I as against 2.5% controls; absorption/imaginative symptoms negatively associated with BD-I; somatoform dissociation associated with number of depressive episodes.
Tekin et al. (40)	Patients with BD, euthymic (*n =* 51), healthy controls (*n =* 49)	DD diagnosis as per DSM-IV criteria; DS severity across groups as rated using DES; association with illness course	35.4% of BD patients qualified for a diagnosis of comorbid DD (depersonalization disorder 17.6%, DD-NOS 15.6%, dissociative amnesia 7.8%, DID 3.9%, dissociative fugue 1.9%); DES scores significantly higher in BD than in controls; DES total score associated with number of suicide attempts and earlier AAO in BD group.
Tuineag et al. (41)	Outpatients with BD-I (*n =* 41), BD-II (*n =* 27) or other BD (*n =* 5)	DS severity as rated using CDS; association with childhood trauma and symptoms of mania, depression and anxiety.	CDS valid for the assessment of DS in BD; CDS total score associated with childhood trauma and symptoms of depression, social anxiety, and panic disorder.
Steardo et al. (42)	Outpatients with BD-I (*n =* 55) and BD-II (*n =* 45)	DS severity as rated using DES-II; association with demographic variables, illness course and treatment response.	DES scores significantly higher in BD-I than in BD-II; DES score significantly associated with number of episodes, presence of mixed or psychotic features, history of suicide attempts or aggressive behavior, symptoms of anxiety, seasonality, antidepressant-induced mania, and poorer treatment response.
Stone et al. (43)	Patients with psychotic BD (*n =* 53) or schizophrenia (*n =* 47), healthy controls (*n =* 51), recruited during the COVID-19 pandemic	DS severity as rated using DES-II; association with childhood trauma and pandemic-related adversities.	DES scores significantly higher in BD and schizophrenia than in healthy controls; significant dissociation (DES > 30) in 17% of BD; no significant association between DES and childhood trauma or pandemic-related adversities.
Li et al. (44)	Inpatients with BD-depression (*n =* 32) and MDD (*n =* 59)	DS severity as rated using CADSS; association with parenting style, betrayal trauma and psychotic symptoms	DS of equal severity in BD and unipolar depression; DS associated with betrayal trauma and severity of psychotic symptoms.

### Characteristics of the Included Studies

The majority of the studies included in this review (*n* = 21) were cross-sectional clinical studies measuring the severity or correlates of DS in patients with BD. Three studies examined the association between syndromal DD and BD (21, 29, 40), while one study each examined associations with cognitive test performance (25) and polymorphisms of specific genes considered to be related to dissociation (23).

### Comorbidity Between BD and DD

Only one study directly measured the frequency of DSM-IV categorical diagnoses of DD in patients with BD. In this study, 35.4% of BD patients fulfilled criteria for one or more DD, with depersonalization disorder (17.6%) being the most frequent (40). A community-based study found that DD were 2.4 times more likely to be diagnosed in patients with mood disorders, but did not distinguish between BD and unipolar depression (21). A study of patients with conversion disorder found a significant association between comorbid diagnoses of DD and BD (35). Finally, a study of discharge records found that 11.4% of patients discharged with BD received a comorbid diagnosis of “anxiety, somatoform or dissociative disorder” as per ICD-9 criteria, but details of individual diagnoses within this group were not reported by the authors (29).

### Presence of Clinically Significant DS in BD

Of the six studies providing estimates of clinically significant DS in BD, as indicated by symptom scores above a specified cut-off, five yielded very similar values in the range of 10–20% (18, 33, 34, 39, 43). A single study yielded a much higher estimate of 51%, but in this study, the control group also reported high levels of DS (24%), suggesting concerns related to methodology or sample selection (28).

### Comparisons of DS Severity Between BD and Other Disorders

Five studies have compared the severity of DS in patients with BD and major depressive disorder (MDD), have measured DS during depressive episodes. In four of these studies, DS were more prominent in BD than in MDD (22, 26, 30, 38), while in the other, they were comparable (44). Studies comparing the severity of DS between BD and other, non-affective psychiatric disorders found that DS were significantly less in BD than in somatoform disorders and DD (18, 19, 32), complex post-traumatic stress disorder (PTSD) (32), atypical psychosis in adolescents (20), and borderline personality disorder (36). DS were comparable in BD and schizophrenia in a single study (43). However, DS scores were significantly higher in patients with BD than in their asymptomatic first-degree relatives (23) and were consistently higher in BD than in healthy controls (28, 33, 34, 37, 39, 43).

### Relationship of DS to BD Subtype

Though some researchers have reported no difference in DS severity scores between BD-I and BD-II (23, 24), there is some evidence that DS and particularly depersonalization symptoms may be more severe in BD-I (30, 42). Only one study assessed DS in patients with other BD subtypes (BD-III and BD not otherwise specified) along with BD-I and BD-II, but the small number of cases in this subgroup precluded a meaningful comparison (41).

### Relationship of DS to Symptomatology in BD

DS scores have been associated with the severity of psychotic symptoms (42, 44); both positive and null results have been reported for associations between DS and depressive symptom severity (31, 41). DS severity has also been associated with general symptom severity across psychopathological dimensions (31), with the severity of symptoms of social anxiety, panic disorder and obsessive-compulsive disorder (33, 41), with the presence of mixed symptoms (42), with suicide attempts (40, 42) and with aggression (42).

### Relationship of DS/DD to Illness Course in BD

Five studies found a negative correlation between the severity of DS and the age at onset of BD (AAO), suggesting an association between dissociation and an early AAO (24, 26, 28, 34, 40). This association appeared to be more specific for depersonalization-related symptoms (24, 26, 34). Only one study reported no association between DS and AAO in BD (38). Associations between DS severity and episode frequency (37), total number of episodes (42) and frequency of manic (25) and depressive episodes (39) have been reported in individual studies. However, a lack of association with episode number has also been reported (38).

### Relationship of DS to Outcome in BD

The severity of DS appears to be associated with treatment response; associations with a higher dose requirement for mood stabilizers (25), with a higher risk of antidepressant-induced mania (42) and with a poorer response to treatment (42) have all been observed. DS are also associated with a poorer quality of life in the “social activities” domain in euthymic BD patients (25).

### Relationship of DS to Other Comorbidities in BD

Higher DS scores have been associated with elevated rates of comorbid obsessive-compulsive disorder (OCD) (22) and panic disorder (24) in BD; no other specific associations with any comorbid diagnosis have been reported.

### Relationship of DS to Environmental Risk Factors in BD

Six studies have examined the association between childhood abuse or neglect and DS in BD; four of these found a positive association between childhood trauma and DS severity (23, 33, 41, 44), while two failed to do so (37, 43). A single study examined the relationship between DS and current stress related to the COVID-19 pandemic, but did not find any significant association between the two (43).

### Relationship of DS to Temperament and Other Psychological Variables in BD

Cyclothymic temperament, considered to be a developmental precursor of BD, was associated with the presence of DS in BD patients in one study (22) but not in another (24). DS severity has also been associated with measures of alexithymia in BD (37).

### Neuropsychological Correlates of DS in BD

A study examining the association between DS and performance on tests of cognition (attention, concentration, executive function and verbal fluency) found no significant association between DS severity and scores on these tests (25).

### Genetic and Biomarker Studies of DS in BD

Only one study has examined the potential genetic correlates of DS in BD; in this study, an interaction between childhood trauma and a functional polymorphism of the *COMT* gene, as well as an additive effect of the *BDNF* gene, was found to predict the severity of DS (23). No other study of any specific biomarker associated with DS/DD in BD has been conducted to date.

## Discussion

Certain features emerge clearly from an overview of the current literature. A significant minority of patients (10–20%) with bipolar disorder experience significant DS, even during the euthymic phase. The overall severity of DS is higher in BD than in healthy controls and in major depression, but is lower in BD when compared with “trauma spectrum disorders” such as DD, complex PTSD and borderline personality disorder. When considering the clinical profile of BD, replicated results suggest that DS are associated with psychotic symptoms, suicide attempts, and a poorer response to treatment. DS also appear to be associated with the severity of childhood trauma in patients with BD. Results related to other symptom domains, episode number and frequency, and quality of life, though of interest, require replication.

The above findings are consistent with the existing literature on DS/DD. Both pathological dissociation and DD are considered part of the “trauma spectrum” of disorders, which are related to exposure to traumatic stress, particularly in childhood (45). This group also includes PTSD and borderline personality disorder; it is perhaps significant that these conditions are also often comorbid with BD (46, 47). Given that childhood adversity is itself a risk factor for BD (48) and was associated with DS in the reviewed studies, this factor may explain a significant proportion of the co-occurrence of DD/DS and BD. Moreover, dissociation is an important mediator of the links between childhood abuse and both psychotic symptoms (49) and suicide (50), which is consistent with the findings observed in patients with BD.

Recent research has shed some light on the neurobiological correlates of dissociative symptoms (51). Some of the replicated biomarkers of pathological dissociation, such as reduced hippocampal and thalamic volumes and elevated peripheral levels of oxytocin, have also been identified in bipolar disorder (52–54), suggesting common neuroanatomical and biochemical substrates for these conditions. It should however be noted that for other biomarkers of dissociation, such as levels of tumor necrosis factor alpha, findings in bipolar disorder are in the opposite direction (55). This suggests that there may be shared mechanistic pathways, but not a complete overlap, between BD and dissociative symptoms.

Besides exposure to childhood trauma or other environmental stressors, the link between DS/DD and BD may be partly mediated through genetic vulnerability. While earlier researchers suggested that this might result from variations in single genes, such as the serotonin transporter (56), more recent results suggest that the overlap between bipolar and dissociative disorders may be polygenic in origin (10).

These findings must be interpreted in the light of important limitations in study design and methodology in the existing literature. The majority of reviewed studies are cross-sectional and focus on clinical variables, with very few studies examining neuropsychological or neurobiological correlates of DD or DS in BD. Most studies have been conducted in remitted, euthymic or clinically stable BD patients, and have measured DS using standardized scales instead diagnosing comorbid DD using standard criteria. There is also substantial heterogeneity in the measurement of DS, with some studies focusing on a subset of DS such as depersonalization/derealization or somatoform dissociation. Sample sizes for BD were generally low (mean: 61.2 ± 47.8), suggesting that some studies may have been underpowered to detect significant differences. Further, in some studies, associations between DS and clinical or environmental variables of interest were not estimated even when the data was available. These factors limit both the value of the conclusions that can be drawn from individual studies and the likelihood of their replication, and suggest the need for better designs even if the research questions are purely clinical in nature.

A further limitation arises from the fact that the link between DS/DD and BD may be non-specific. Dissociative symptoms of severity comparable to or slightly greater than those reported in BD have been observed in a wide range of psychiatric disorders, including schizophrenia, anxiety disorders, eating disorders and substance use disorders (57). These findings suggest that dissociation may be better considered from a dimensional rather than a categorical perspective, or that it may be related to a common genetic substrate that cuts across traditional psychiatric diagnoses (58).

Despite these limitations, the above review suggests that the presence of DS/DD in patients with BD may have significant clinical and research implications. From a clinical perspective, practitioners should be aware of the co-occurrence of these conditions, and maintain a high index of clinical suspicion; in cases of doubt, a standardized rating scale such as the DES can aid decision-making. Given the replicated associations with psychotic symptoms, suicidality, and poor treatment response, these patients may require more intensive clinical management. Lithium therapy, which has been shown to reverse the hippocampal and thalamic volume reductions common to bipolar disorder and dissociation (51, 52), may be a useful therapeutic option. Bipolar patients with DS/DD should be screened for other comorbid anxiety disorders as well as post-traumatic stress disorder (22, 24, 33, 41). Given the link between childhood trauma and dissociation in BD, a sensitive inquiry into possible childhood abuse or neglect should be made when patients are clinically stable. Finally, when there are significant DS or a syndromal DD, appropriate psychological interventions should be provided (59).

From a research perspective, the following areas require particular attention in the study of the links between dissociation and bipolarity:

- Replications of findings related to clinical and psychological variables of interest, such as affective temperaments, alexithymia, cycle length and the presence of mixed features
- Accurate studies of the prevalence of comorbid DD in BD, and of comorbid BD in DD, using standard diagnostic criteria
- Longitudinal studies of the impact of DS or DD on the course and outcome of BD
- Studies of high-risk youth (e.g., with a family history of BD or with a history of childhood abuse) with DS or DD, to assess their subsequent risk of BD and the possibility of early intervention (12, 60)
- Studies of structural, functional and biochemical markers of the link between BD and DD; areas that could be immediately explored include associations with peripheral levels of cytokines (61) and functional brain imaging studies focusing on key frontal and subcortical regions implicated in both disorders (49, 62)
- Assessment of the utility of a dimensional rather than a categorical approach to the study of dissociation in patients with BD, and of the correlations between DS and other symptom dimensions in BD (63)
- Genome-wide association studies using either a narrower definition of DD (i.e., without lumping them with anxiety and somatoform disorders) or a continuous measure of DS, to identify specific and shared genetic loci associated with vulnerability to pathological dissociation in patients with BD
- Evaluation of the efficacy of specific pharmacological (mood stabilizer, antipsychotic), brain stimulation (rTMS) and psychotherapeutic (trauma-focused) approaches in patients with BD and DD/DS (64, 65).

## Conclusion

Though research on dissociative symptoms and disorders in patients with bipolar disorders is still in its infancy, existing evidence suggests that these symptoms are significantly associated with both risk factors—particularly childhood abuse—and a specific illness profile in bipolar disorder. It is hoped that the findings reviewed and summarized above will be of use to clinicians working with patients with bipolar disorder. Moreover, the tentative research agenda outlined above could improve our understanding of this specific comorbidity, leading to improved strategies for early intervention as well as treatment in subsequent stages of bipolar disorder.

## Author Contributions

RR selected the review topic and method, conducted the literature search and article selection, analyzed and summarized the results, and wrote and edited the manuscript.

## Conflict of Interest

The author declares that the research was conducted in the absence of any commercial or financial relationships that could be construed as a potential conflict of interest.

## Publisher's Note

All claims expressed in this article are solely those of the authors and do not necessarily represent those of their affiliated organizations, or those of the publisher, the editors and the reviewers. Any product that may be evaluated in this article, or claim that may be made by its manufacturer, is not guaranteed or endorsed by the publisher.

## Abbreviations

AAO: age at onset
BD: bipolar disorders
BD-I: bipolar I disorder
BD-II: bipolar II disorder
BDNF: brain-derived neurotrophic factor
CADSS: Clinician-Administered Dissociative States Scale
CDS: Cambridge Depersonalization Scale
COMT: catechol O-methyltransferase
DAT: dopamine transporter
DID: dissociative identity disorder
DRD4: dopamine type 4 receptor
DD: dissociative disorders
DD-NOS: dissociative disorder not otherwise specified
DES: Dissociative Experiences Scale
DSM-IV: Diagnostic and Statistical Manual for Mental Disorders, Fourth Edition
HAM-D: Hamilton Rating Scale for Depression
MDD: major depressive disorder
OCD: obsessive-compulsive disorder
PTSD: post-traumatic stress disorder
SCI-DER: Structured Clinical Interview for Depersonalization/Derealization Spectrum
SCL-90-R: Symptom Checkist-90 Revised
SDQ-20: Somatoform Dissociation Questionnaire-20
SERT: serotonin transporter.
